# Supplementary material for: Pattern separation of spiketrains in hippocampal neurons
Source: Sci Rep. 2019 Mar 27;9:5282. doi: 10.1038/s41598-019-41503-8 (PMC6437159; doi:10.1038/s41598-019-41503-8)
Supplement: Supplementary file 1 — Supplements [file 41598_2019_41503_MOESM1_ESM.pdf]

Supplements

Tables and supplementary figures for "Pattern separation of spiketrains in hippocampal neurons"

Authors: A.D. Madar, L.A. Ewell, M.V. Jones.

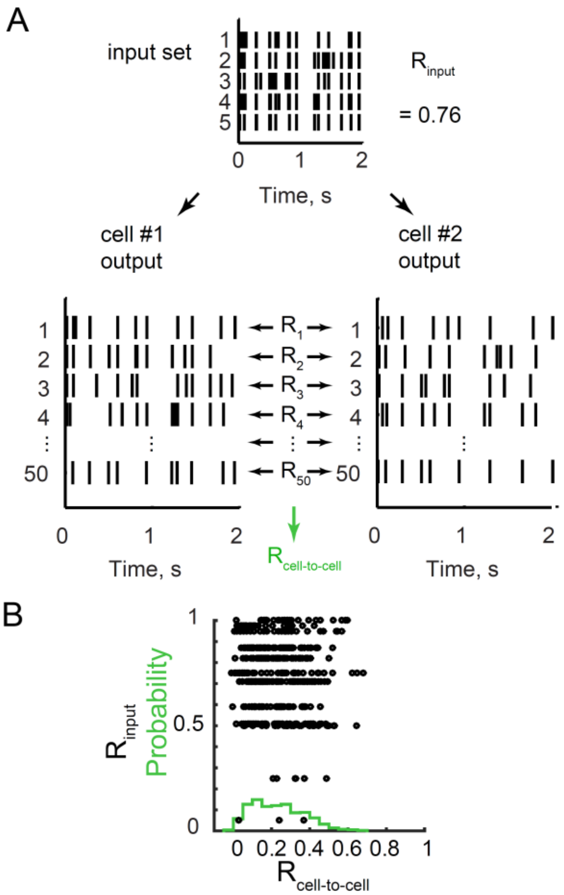

Figure S1. Different granule cells process identical inputs differently.
(A) The similarity between pairs of spiketrains coming from two different output sets but associated to the same input set and with the same sweep number is assessed with the Pearson's correlation coefficient (τ_w = 10 ms). The fifty resulting coefficients are then averaged to give $R_{cell\text{-}to\text{-}cell}$, a single number measuring the overall similarity of all output spiketrains between two output sets. **(B)** Probability distribution of $R_{cell\text{-}to\text{-}cell}$ (green histogram) across all GC recordings (all combinations of pairs of GC output sets from the same input set were compared). The distribution of $R_{cell\text{-}to\text{-}cell}$ (black circles) is not dependent on R_{input}.

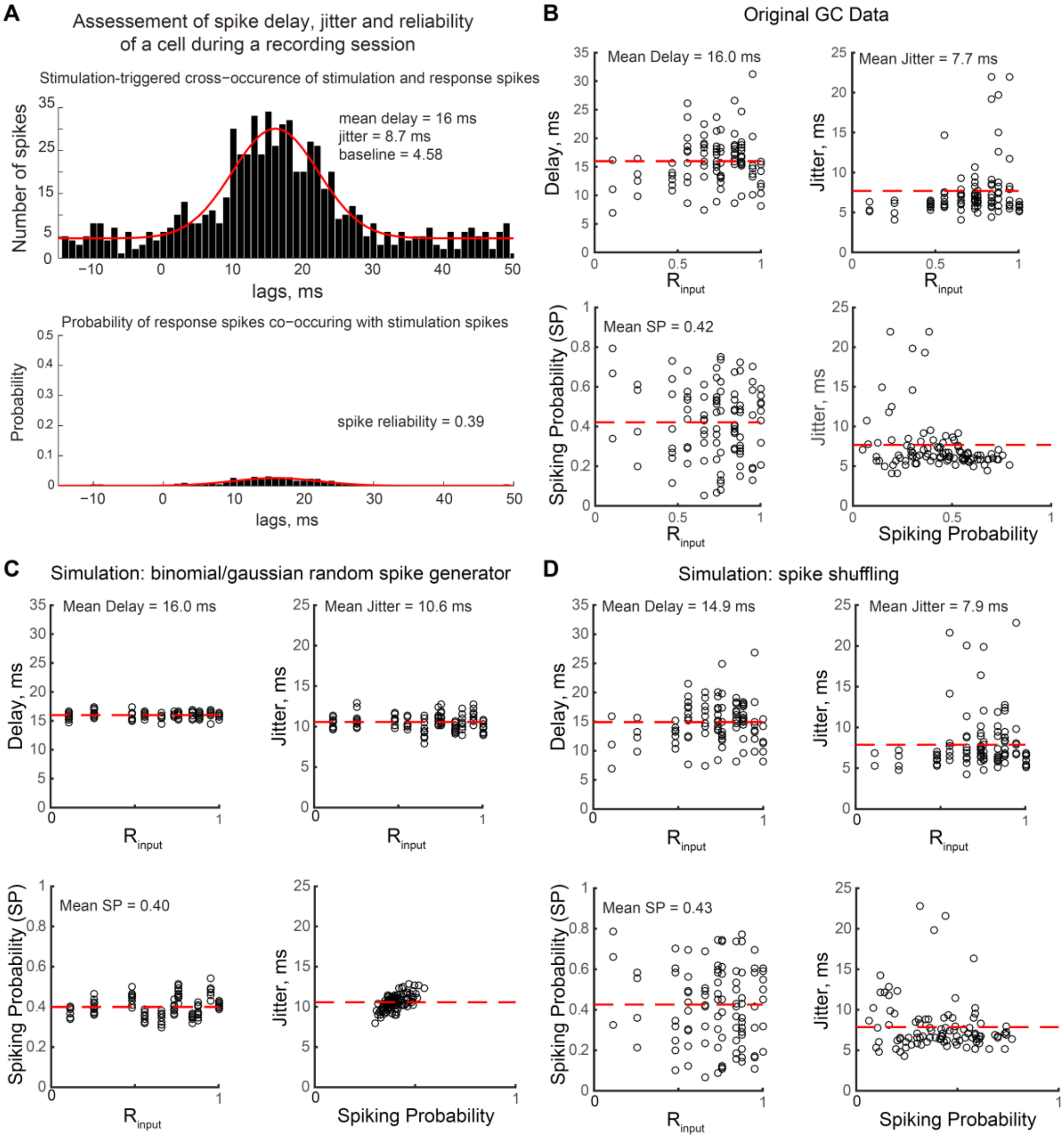

Figure S2 related to Figure 7. Spike delay, jitter and reliability distributions for real data, simulations and shuffled data

(A) Cross-occurrence method to measure spike delay, jitter and spiking reliability of a neuron during a given recording session. *Top*: Example histogram of output spikes occurring after input spikes, fitted (red curve) with a Gaussian distribution $N(\mu,\sigma, \text{baseline})$, where μ is the mean delay and σ is the jitter of this delay. Lag 0 ms corresponds to the input spike time. In this example, output spikes are generated on average 16 ms after a stimulation impulse (delay) with a jitter (σ) of 8.7 ms. *Bottom***:** the baseline is subtracted and the histogram divided by the number of input spikes during the recording session. This gives the distribution of the probability of spiking after an input spike, the sum of probabilities defining the spiking probability (SP) of the cell during the recording session. Here the neuron fires 39 % of the time after an input spike.

(B-D) Delay, jitter and spiking probability (SP) distributions as a function of input sets, for (**B**) the original GC recordings, (**C**) the simulations of binomially random spiking with Gaussian delay (n = 110) and (**D**) one spike shuffling dataset (n = 102). Dashed horizontal red lines are means.

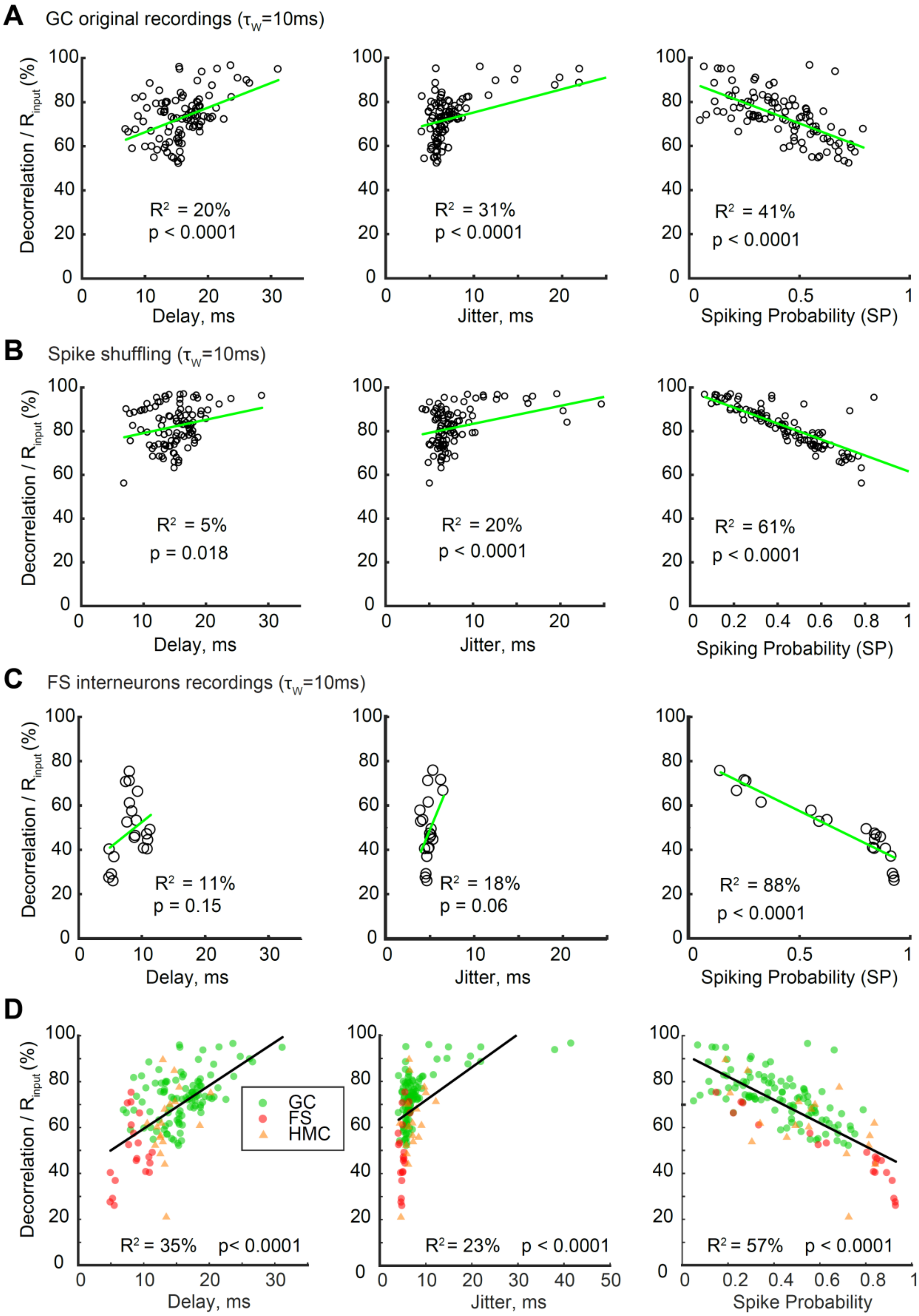
A
GC original recordings (τW=10ms)
Decorrelation / Rinput (%)
Delay, ms
Jitter, ms
Spiking Probability (SP)
R² = 20%
p < 0.0001
R² = 31%
p < 0.0001
R² = 41%
p < 0.0001
B
Spike shuffling (τW=10ms)
R² = 5%
p = 0.018
R² = 20%
p < 0.0001
R² = 61%
p < 0.0001
C
FS interneurons recordings (τW=10ms)
R² = 11%
p = 0.15
R² = 18%
p = 0.06
R² = 88%
p < 0.0001
D
GC
FS
HMC
R² = 35% p< 0.0001
R² = 23% p < 0.0001
R² = 57% p < 0.0001
Spike Probability

Figure S3 related to Figure 7. Spike-wise neural noise characteristics are not good predictors of spiketrain decorrelation by single GCs.

Plots of the normalized decorrelation, i.e. $(R_{input} – R_{output}) / R_{input}$, of each recording set (τ_w = 10 ms) : 102 for GC original and shuffled recordings **(A-B)**, 20 for FS **(C)**, and for GC, FS and HMC pooled together (**D**) as a function of spike-wise noise characteristics (spike delay, jitter and probability). Solid green lines are the best linear fit, with R^2 and p-values noted in each panel. These plots illustrate **Table S2**. Note that decorrelation is poorly explained (low R^2) by either the spike delay or its jitter in all cell-types. In contrast, the spiking probability (SP) is a good predictor of decorrelation in shuffled GC recordings (n = 102 recordings entirely dominated by spike-wise noise. See Figure S2) and even more so in FS recordings (for FS, SP was computed from nbFS data. See Figure S7). This suggests that a low SP can be a potent mechanism for decorrelation, and that FS show different levels of decorrelation than GCs partly because they are more reliable. However, the regression line for FS is lower than for GCs (even FS with low SP show less decorrelation than GCs with similar SP), and SP is only an average predictor of decorrelation for GCs, thus confirming that temporal pattern separation in single GCs cannot be the result of simple neural noise.

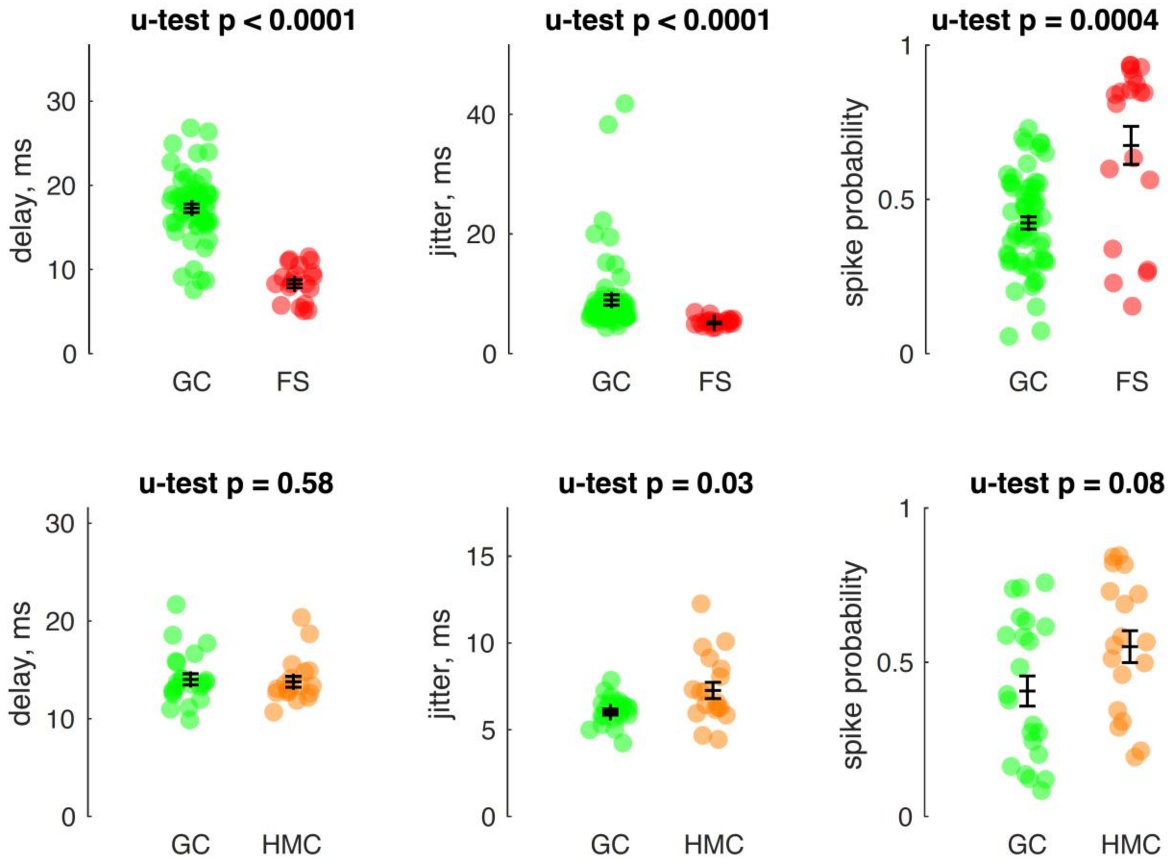

Figure S4 related to Figure 7. Spike-wise noise characteristics of granule cells are different than those of DG interneurons.
Mean +/- SEM (bars) and individual recordings (dots). Spike delay, jitter and spiking probability (SP) for FS interneurons compared to GC recordings associated with the same input sets than FS (*top*) (20 FS recordings, 61 GC recordings, see Figure 3A) and HMC interneurons compared to a different set of GC recordings associated with the same input sets than HMC (*bottom*) (18 HMC recordings, 22 GC recordings, see Figure 3B). A U-test was applied to each pair of comparison, showing that FS are much faster and less noisy than GCs, whereas HMC are similar to GCs (slightly larger jitter, but slightly higher SP).

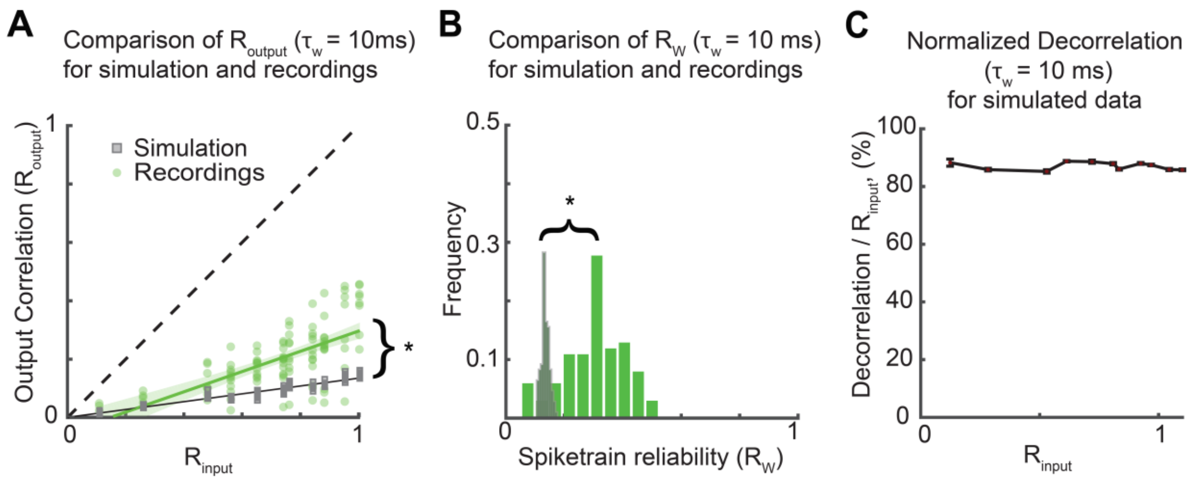

Figure S5 related to Figure 7. R_{output} and spiketrain reliability are lower for a random spike generator than for GCs.

A Binomial/Gaussian random spike generator with parameters based on the mean spike-wise neural noise measured in GCs leads to more decorrelation but less spiketrain reliability than in GCs.

(A) R_{output} distribution at $\tau_w = 10$ ms, for simulated and GC recordings. (ANCOVA: $p < 0.0001$). Shaded areas (green and grey) represent the 95% CI of the regressions.

(B) R_w distributions are significantly different (unpaired T-test correcting for unequal variances: $p < 0.0001$, <Rw>simul = 0.14).

(C) Like in the original data (**Fig 2E**), the average normalized decorrelation $((R_{input}-R_{output})/R_{input})$ seems invariant. Bars are SEM.

(A-B) Asterisks: $p < 0.05$.

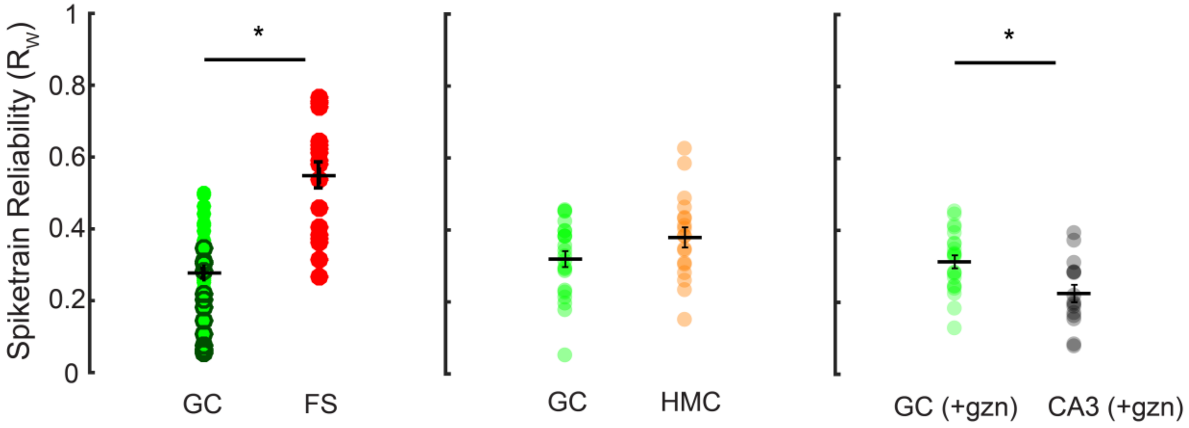

Figure S6 related to Figure 8A. Spiketrain reliability in different hippocampal celltypes

FS have a higher R_w than GCs (n = 20 vs 61 recording sets, unpaired T-test: $p < 0.0001$). Note that when comparing only the simultaneous FS and GC recordings (dark green), we found a similarly significant difference. HMCs R_w are not significantly higher than in GCs (n = 18 vs 22 recording sets, unpaired T-test: $p = 0.0963$). Under partial block of inhibition, CA3 pyramidal cells have a lower R_w than GCs (n = 15 vs 22 recording sets, unpaired T-test: $p = 0.0052$). Asterisks: $p < 0.05$

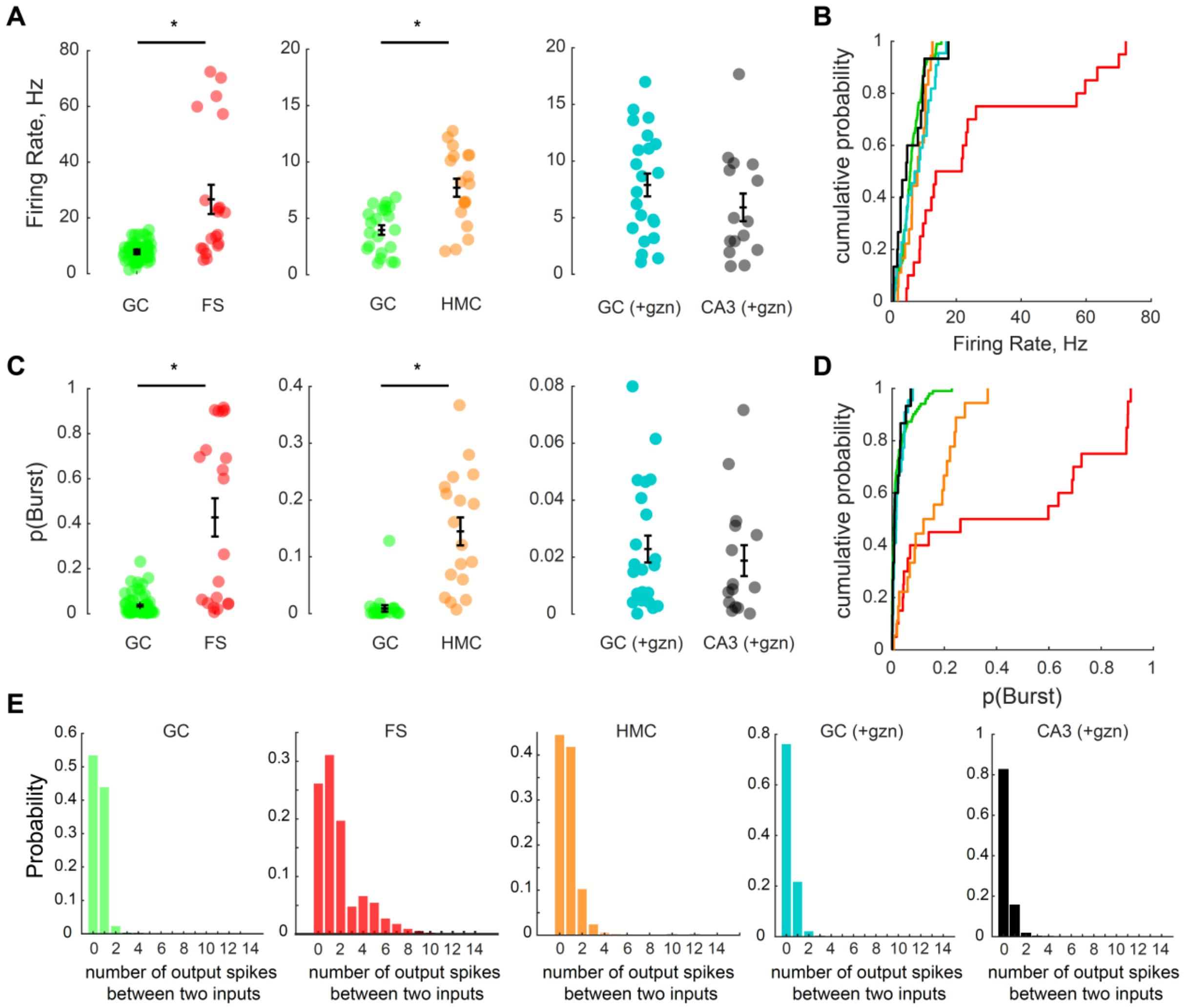

Figure S7 related to Figure 3. DG interneurons fire bursts and have a higher firing rate than GCs

(A-B) Comparison of the firing rate in all celltypes and conditions. **(A)** For each comparison the GC dataset is different and matched to the non-GC dataset, as in Figure 3-4. FS and HMC have a higher firing rate than their GC control (U-test: $p < 0.0001$ and $p = 0.0005$ respectively) whereas CA3 and GC recordings under gabazine and 30 Hz input trains do not differ (U-test: $p = 0.18$).

(B) Cumulative distributions of the firing rate for all recordings of all celltypes (n = 102 GC, 20 FS, 18 HMC, 15 CA3+gzn, 22 GC+gzn).

(C-D) Same as A-B for the probability of bursting in a recording. Bursting was defined as the occurrence of at least two action potentials between two input pulses. **(C)** FS and HMC have a higher propensity to fire bursts than their GC control (U-test: $p < 0.0001$) whereas CA3 pyramidal cells and their GC control do not differ (U-test: $p = 0.74$).

(E) The distribution of the number of spikes between two input pulses, for all celltypes, show that FS are the only neurons that consistently fire large bursts. Note that GCs almost never fire more than twice, and when they do it generally corresponds to two preceding input pulses occurring close to each other (the first spike being in reality associated to a different input than the next spike).

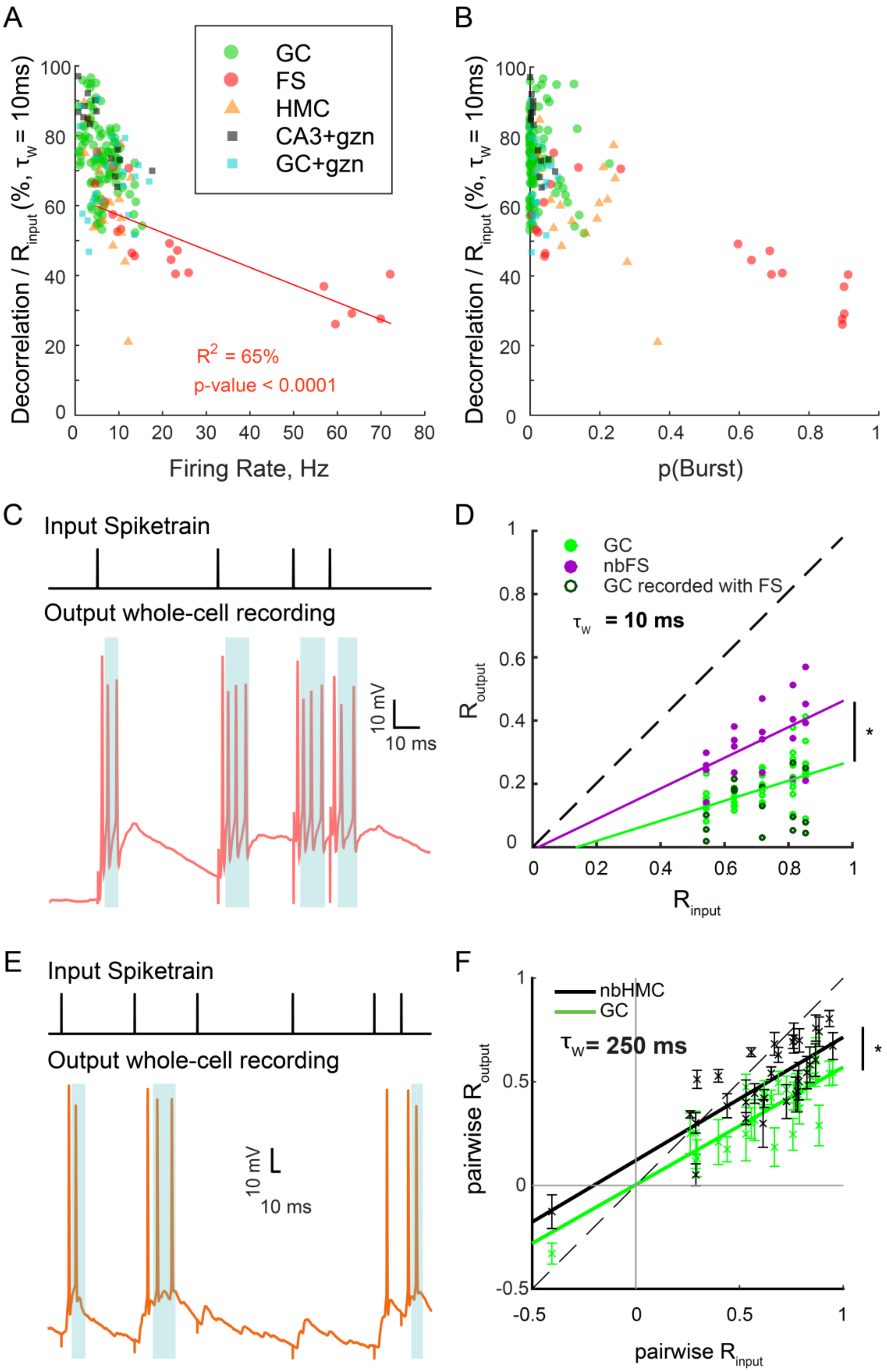
A
GC
FS
HMC
CA3+gzn
GC+gzn
Decorrelation / R_{input} (%, τ_W = 10ms)
R^2 = 65%
p-value < 0.0001
Firing Rate, Hz
B
p(Burst)
C
Input Spiketrain
Output whole-cell recording
10 mV
10 ms
D
GC
nbFS
GC recorded with FS
τ_W = 10 ms
R_{output}
R_{input}
*
E
Input Spiketrain
Output whole-cell recording
10 mV
10 ms
F
nbHMC
GC
τ_W= 250 ms
pairwise R_{output}
pairwise R_{input}
*

96

Figure S8 related to Figure 3 and 5. Differences in pattern separation between GC and DG interneurons are not solely due to different firing rates and bursting behaviors.

(A) The relationship between the firing rate and decorrelation levels in all recordings of all celltypes. In contrast to GCs, for FS neurons there is a strong correlation between the firing rate of a recording set and the associated normalized decorrelation. See **Table S3** for more details.

(B) The relationship between the probability of bursting (see Figure S7) and the decorrelation levels in all recordings of all celltypes is less clear, but cells bursting more than 30% of the time have the lowest decorrelation levels.

(C) Example of bursts in a FS recording (*bottom*) in response to input spikes (*top*). To assess the effect of bursting on R_{output}, we truncated each recorded spiketrain from FS neurons to keep only the first output spike between two input spikes, thus removing any burst without altering the SP of the cell. The blue shaded areas highlight the spikes that were removed. The resulting truncated dataset was termed "nbFS" for "non-burst FS".

(D) R_{output} versus R_{input} for nbFS and GC at τ_w = 10ms (to compare to Figure 3A4). Both distributions are still significantly different, suggesting the bursting behavior of FS is not sufficient to explain the difference in pattern separation: ANCOVA: $p < 0.0001$.

(E) Bursts in HMC recordings were truncated to produce an "nbHMC" dataset.

(F) Pairwise analysis on nbHMC and GC recordings at τ_w = 250 ms show that the distributions are still different between the two celltypes (to compare to Figure 5D): ANCOVA: $p < 0.0001$. Under this analysis, nbHMCs and GCs are also significantly different at lower timescales (τ_w = 100, 50 and 10 ms) but with a lower effect size as τ_w decreases (not shown).

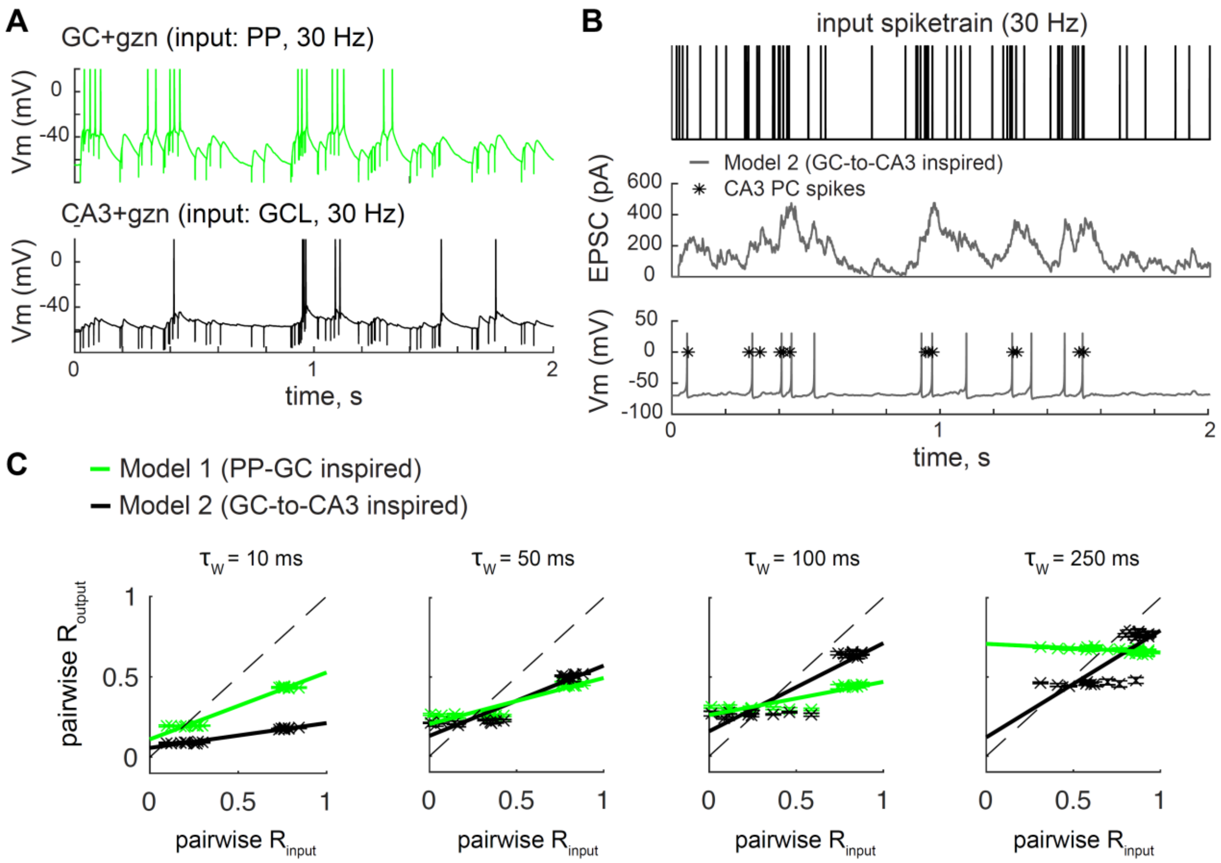

Figure S9 related to Figure 5 and 9. Short-term synaptic dynamics differences can drive differences in terms of temporal pattern separation.

(A) Examples of current-clamp recordings in one GC and one CA3 PC, under partial block of inhibition, in response to a 30 Hz input train (input pulses are noticeable as downward artefacts). EPSPs visually appear quite different between GCs and CA3 PCs, with clear facilitation in CA3 PCs, which leads them to spike mostly during periods of high input frequencies.

(B-C) We designed two models of spiking neurons only differing by a few synaptic parameters. Model 1 is the same as the model presented in Figure 9, with depressing EPSC dynamics, except that the inhibition constant was decreased to match FR observed in real GC+gzn recordings. Model 2 was inspired by GC-to-CA3 mossy fiber synapses that exhibit low initial probability of release and short-term facilitation, and the inhibition constant was set so to match the mean FR of real CA3+gzn recordings (~7Hz). Model 1 and 2 were thus designed to have different synaptic dynamics but yield similar FR. Spiking responses to the pattern separation protocols used on real CA3 PCs and their GC controls (30 Hz input trains: R_{input} = 0.21 and 0.76, shown in Figure 4) were then simulated (n = 5 simulated output sets for each model and each R_{input}).

(B) Example of current and voltage responses of model 2 to a 30 Hz input train. Asterisks correspond to spike times of a single sweep from a CA3 PC recording (different than in A).

(C) Pairwise correlation analysis, as in Figure 5, shows that the two models yield visually obvious differences in terms of temporal pattern separation (crosses and bars: mean +/- SEM; solid lines: linear regression on data points). Although model 1 and 2 do not reproduce well the pattern separation levels observed in real GCs and CA3 PCs (Figure 5E), they qualitatively go in the same direction: the GC-to-CA3 inspired model 2 produces visually lower R_{output} than the PP-GC inspired model 1, at short timescales (10 ms) and large ones (250 ms, for low R_{input} values). Overall, this analysis shows that differences in terms of synaptic dynamics are sufficient to cause differences in terms of temporal pattern separation.

Table S1-3. **Linear regressions goodness-of-fit, p-value and slope.** The predictor variables (x-axis) correspond to columns, and the variables to be explained (y-axis) correspond to rows. Red highlights significant regressions that explain more than 50% of the variance ($R^2 > 50\%$). Blue highlights regressions that are significant ($p < 0.01$) but that explain less than 50% of the variance. The values used for Normalized Decorrelation, i.e. $(R_{input} - R_{output})/ R_{input}$, and for Spiketrain Reliability (R_w) were computed with a binning window of 10 ms, unless specified. Abbreviations: GC yo = from young mice, GC ad = from adult mice, ALL = dataset pooling all celltypes and conditions recorded in young mice

Table S1. Intrinsic electrophysiological cell properties

y-axis ↓ / x-axis →		Membrane Capacitance (Cm)	Membrane Resistance (Rm)	Membrane Time Constant = Rm.Cm	Resting Membrane Potential (Vrest)
Normalized Decorrelation	GC yo	R^2 = 4%<br>p = 0.08<br>slope = -0.2<br>F(1,100) = 3.2	R^2 = 5%<br>p = 0.06<br>slope = -0.03<br>F(1,100) = 3.7	R^2 = 8%<br>p = 0.013<br>slope = -1.2<br>F(1,100) = 6.5	R^2 = 3%<br>p = 0.17<br>slope = -0.2<br>F(1,100) = 1.9
	FS	R^2 = 47%<br>p = 0.0008<br>slope = -1.5<br>F(1,18) = 15.8	R^2 = 77%<br>p <0.0001<br>slope = 0.6<br>F(1,18) = 58.9	R^2 = 5%<br>p = 0.4<br>slope = 13.6<br>F(1,18) = 1.2	R^2 = 46%<br>p = 0.0009<br>slope = 1.4<br>F(1,18) = 15.6
	HMC	R^2 = 33%<br>p = 0.013<br>slope = -0.4<br>F(1,16) = 7.8	R^2 = 3%<br>p = 0.48<br>slope = 0.06<br>F(1,16) = 0.5	R^2 = 7%<br>p = 0.3<br>slope = -1.8<br>F(1,16) = 1.6	R^2 = 0.5%<br>p = 0.8<br>slope = 0.1<br>F(1,16) = 0.08
	CA3 +gzn	R^2 = 0.1%<br>p = 0.9<br>slope = -0.05<br>F(1,13) = 0.01	R^2 = 2%<br>p = 0.59<br>slope = 0.03<br>F(1,13) = 0.3	R^2 = 4%<br>p = 0.60<br>slope = 1.2<br>F(1,13) = 0.6	R^2 = 29%<br>p = 0.04<br>slope = 0.7<br>F(1,13) = 5.4
	GC +gzn	R^2 = 30%<br>p = 0.008<br>slope = 1.6<br>F(1,20) = 8.6	R^2 = 0.3%<br>p = 0.8<br>slope = 0.01<br>F(1,20) = 0.06	R^2 = 2%<br>p = 0.5<br>slope = 3.5<br>F(1,20) = 4.1	R^2 = 13%<br>p = 0.09<br>slope = -0.7<br>F(1,20) = 3.1
	ALL	R^2 = 0.6%<br>p = 0.35<br>slope = -0.09<br>F(1,175) = 0.9	R^2 = 3%<br>p = 0.04<br>slope = 0.03<br>F(1,175) = 4.2	R^2 = 1%<br>p = 0.2<br>slope = 0.55<br>F(1,175) = 1.5	R^2 = 0.2%<br>p = 0.55<br>slope = -0.08<br>F(1,175) = 0.3
	GC ad	R^2 = 4%<br>p = 0.24<br>slope = -0.4<br>F(1,33) = 1.4	R^2 = 11%<br>p = 0.06<br>slope = -0.07<br>F(1,33) = 3.9	R^2 = 17%<br>p = 0.015<br>slope = -4.3<br>F(1,33) = 6.6	R^2 = 5%<br>p = 0.2<br>slope = -0.3<br>F(1,33) = 1.7
Spiketrain Reliability (R_W)	GC yo	R^2 = 2%<br>p =0.2<br>slope = 0.001<br>F(1,100) = 1.8	R^2 = 6%<br>p = 0.03<br>slope = 4e-3<br>F(1,100) = 4.7	R^2 = 7%<br>p = 0.02<br>slope = 0.01<br>F(1,100) = 6.1	R^2 = 3%<br>p = 0.17<br>slope = 0.002<br>F(1,100) = 1.9
	FS	R^2 = 48%<br>p =0.0006, slope = 0.01<br>F(1,18) = 16.8	R^2 = 70%<br>p < 0.0001<br>slope = -0.007<br>F(1,18) = 41.7	R^2 = 6%<br>p = 0.29<br>slope = -0.12<br>F(1,18) = 0.7	R^2 = 39%<br>p = 0.003<br>slope = -0.014<br>F(1,18) = 11.6
	HMC	R^2 = 29%<br>p = 0.19<br>slope = 0.005<br>F(1,16) = 6.5	R^2 = 24%<br>p = 0.06<br>slope = -0.001<br>F(1,16) = 5.0	R^2 = 9%<br>p = 0.2<br>slope = 0.006<br>F(1,16) = 0.3	R^2 = 3%<br>p = 0.5<br>slope = -0.002<br>F(1,16) = 0.5
	CA3 +gzn	R^2 = 4%<br>p = 0.5<br>slope = 0.003<br>F(1,13) = 0.5	R^2 = 9%<br>p = 0.29<br>slope = 6e-4<br>F(1,13) = 1.2	R^2 = 4%<br>p = 0.45<br>slope = -0.01<br>F(1,13) = 0.45	R^2 = 10%<br>p = 0.24<br>slope = -0.004<br>F(1,13) = 1.5
	GC +gzn	R^2 = 2%<br>p = 0.5<br>slope = -0.003<br>F(1,20) = 0.4	R^2 = 22%<br>p = 0.03<br>slope = 8e-4<br>F(1,20) = 5.7	R^2 = 17%<br>p = 0.05<br>slope = -0.02<br>F(1,20) = 3.9	R^2 = 29%<br>p = 0.01<br>slope = 0.008<br>F(1,20) = 8.0
	ALL	R^2 = 0.4%<br>p = 0.5<br>slope = 7e-4<br>F(1,175) = 0.5	R^2 = 7%<br>p = 0.0013<br>slope = -4e-4<br>F(1,175) = 10.7	R^2 = 3%<br>p = 0.045<br>slope = -0.008<br>F(1,175) = 4.1	R^2 = 1%<br>p = 0.24<br>slope = 0.001<br>F(1,175) = 1.4
	GC ad	R^2 = 13%<br>p =0.03<br>slope = 0.006<br>F(1,33) = 4.9	R^2 = 6%<br>p = 0.17<br>slope = 4e-4<br>F(1,33) = 2.0	R^2 = 24 %<br>p = 0.003<br>slope = 0.04<br>F(1,33) = 10.3	R^2 = 8.5%<br>p = 0.09<br>slope = 0.004<br>F(1,33) = 3.05

Table S2. Spike-wise neural noise

y-axis ↓ / x-axis →		Delay	Jitter	Spike Probability (SP)
Normalized Decorrelation	GC yo	R^2 = 20%<br>p < 0.0001<br>slope = 1.1<br>F(1,100) = 24.5	R^2 = 31%<br>p < 0.0001<br>slope = 1.05<br>F(1,100) = 45.1	R^2 = 41%<br>p < 0.0001<br>slope = -37.6<br>F(1,100) = 69.8
	FS	R^2 = 11%<br>p = 0.15<br>slope = 2.3<br>F(1,18) = 2.2	R^2 = 18%<br>p = 0.06<br>slope = 9.6<br>F(1,18) = 3.9	R^2 = 88%<br>p < 0.0001<br>slope = -49.1<br>F(1,18) = 129.8
	HMC	R^2 = 9%<br>p = 0.22<br>slope = 2.1<br>F(1,16) = 1.6	R^2 = 18%<br>p = 0.08<br>slope = 3.4<br>F(1,16) = 3.5	R^2 = 34%<br>p = 0.011<br>slope = -42.0<br>F(1,16) = 8.1
	ALL	R^2 = 35%<br>p < 0.0001<br>slope = 1.9<br>F(1,175) = 74.3	R^2 = 23%<br>p < 0.0001<br>slope = 1.4<br>F(1,175) = 41.0	R^2 = 57%<br>p < 0.0001<br>slope = -50.6<br>F(1,175) = 183.8
	GC ad	R^2 = 2%<br>p = 0.38<br>slope = 0.9<br>F(1,33) = 0.8	R^2 = 4%<br>p = 0.23<br>slope = -3.5<br>F(1,33) = 1.4	R^2 = 42%<br>p < 0.0001<br>slope = -45.7<br>F(1,33) = 23.8
	Shuffle (GC yo)	R^2 = 5%<br>p = 0.02<br>slope = 0.6<br>F(1,100) = 5.8	R^2 = 20%<br>p < 0.0001<br>slope = 0.8<br>F(1,100) = 25.6	R^2 = 61%<br>p < 0.0001<br>slope = -36.5<br>F(1,100) = 160.0
Spiketrain Reliability (R_W)	GC yo	R^2 = 23%<br>p < 0.0001<br>slope = -0.012<br>F(1,100) = 29.9	R^2 = 37%<br>p < 0.0001<br>slope = -0.012<br>F(1,100) = 58.9	R^2 = 48%<br>p < 0.0001<br>slope = 0.42<br>94.1
	FS	R^2 = 10%<br>p = 0.16<br>slope = -0.025<br>F(1,18) = 2.1	R^2 = 11%<br>p = 0.15<br>slope = -0.084<br>F(1,18) = 2.2	R^2 = 87%<br>p < 0.0001<br>slope = 0.55<br>F(1,18) = 117.7
	HMC	R^2 = 36%<br>p = 0.009<br>slope = -0.030<br>F(1,16) = 8.8	R^2 = 53%<br>p = 0.0007<br>slope = -0.043<br>F(1,16) = 17.7	R^2 = 31%<br>p = 0.016<br>slope = 0.30<br>F(1,16) = 7.3
	ALL	R^2 = 40%<br>p < 0.0001<br>slope = -0.020<br>F(1,175) = 93.4	R^2 = 28%<br>p < 0.0001<br>slope = -0.016<br>F(1,175) = 53.4	R^2 = 61%<br>p < 0.0001<br>slope = 0.5<br>F(1,175) = 219.1
	GC ad	R^2 = 7%<br>p = 0.14<br>slope = -0.01<br>F(1,33) = 2.3	R^2 = 0.03%<br>p = 0.9<br>slope = 0.003<br>F(1,33) = 0.01	R^2 = 16%<br>p = 0.017<br>slope = 0.2<br>F(1,33) = 6.3
	Shuffle (GC yo)	R^2 = 5%<br>p = 0.03<br>slope = -0.006<br>F(1,100) = 5.0	R^2 = 21%<br>p < 0.0001<br>slope = -0.008<br>F(1,100) = 26.2	R^2 = 62%<br>p < 0.0001<br>slope = 0.4<br>F(1,100) = 162

Table S3. Spiketrain-wise properties

y-axis ↓ / x-axis →		**Overall Firing Rate**	**Spiketrain Reliability (R_W)**
**Normalized Decorrelation**	**GC yo**	R^2 = 15%<br>p < 0.0001<br>slope = - 1.3<br>F(1,100) = 18.4	R^2 = 81%<br>p < 0.0001<br>slope = -87<br>F(1,100) = 430.1
	**FS**	R^2 = 65%<br>p < 0.0001<br>slope = - 0.5<br>F(1,18) = 33.5	R^2 = 90%<br>p < 0.0001<br>slope = -85<br>F(1,18) = 169.0
	**HMC**	R^2 = 35%<br>p = 0.0010<br>slope = - 2.8<br>F(1,16) = 8.6	R^2 = 61%<br>p = 0.0001<br>slope = -105<br>F(1,16) = 25.5
	**CA3 +gzn**	R^2 = 69%<br>p = 0.0001<br>slope = - 1.7<br>F(1,13) = 28.7	R^2 = 55%<br>p = 0.0016<br>slope = -76<br>F(1,13) = 15.8
	**GC +gzn**	R^2 = 0.6%<br>p = 0.73<br>slope = - 0.2<br>F(1,20) = 0.1	R^2 = 28%<br>p = 0.0111<br>slope = -75<br>F(1,20) = 7.8
	**ALL**	R^2 = 37%<br>p < 0.0001<br>slope = - 0.8<br>F(1,175) = 101.5	R^2 = 79%<br>p < 0.0001<br>slope = -91<br>F(1,175) = 673.1
	**GC ad**	R^2 = 30%<br>p = 0.0006<br>slope = - 4.1<br>F(1,33) = 14.5	R^2 = 52%<br>p < 0.0001<br>slope = -90<br>F(1,33) = 36.2

Table S4. Statistics

Figure	Test	N		Descriptive stats	p-value	Degrees of freedom & F/t/z/R/etc value	other
2d	One-sample T-test	8<br>8<br>13<br>13<br>10<br>13<br>11<br>11<br>8<br>4<br>3	recorded neurons	mean +/- SEM.	< 0.0001<br>< 0.0001<br>< 0.0001<br>< 0.0001<br>< 0.0001<br>< 0.0001<br>< 0.0001<br>< 0.0001<br>< 0.0001<br>0.0002<br>0.0093	t(7) = 21.81<br>t(7) = 12.81<br>t(12) = 22.70<br>t(12) = 24.49<br>t(9) = 19.91<br>t(12) = 33.62<br>t(10) = 41.62<br>t(10) = 24.15<br>t(7) = 20.56<br>t(3) = 22.28<br>t(2) = 10.325	Rinput =<br>1<br>0.95<br>0.88<br>0.84<br>0.76<br>0.74<br>0.65<br>0.56<br>0.47<br>0.26<br>0.11
2d	Unbalanced one-way ANOVA	8, 8, 13, 13, 10, 13, 11, 11, 8, 4, 3	11 groups of 102 recording sets from 28 neurons	mean +/- SEM	< 0.0001	F(10,91) = 30.05	
2d	Post-hoc Tukey-Kramer Multiple-comparison	8, 8, 13, 13, 10, 13, 11, 11, 8, 4, 3	recorded neurons	mean +/- SEM	see Fig2d right		
2e	unbalanced	8, 8,	11 groups	mean +/- SEM	0.19	F(10,91) = 1.42	

	one-way ANOVA	13, 13, 10, 13, 11, 11, 8, 4, 3	of 102 recording sets (see above)				
3A4	ANCOVA	20, 61	recording sets (FS, GC)	Linear best fit, 95% CI	< 0.0001	F(2,77) = 38.70	
3A4	Unbalanced two-way ANOVA	20, 61	recording sets (FS, GC)		0.016<br>< 0.0001<br>0.72	F(4,71) = 4.86<br>F(1,71) = 69.65<br>F(4,71) = 0.52	Input groups<br>Cell types<br>Interaction
3A4	Post-hoc two-sample T-test with Bonferroni correction for 5 comparison groups	4, 13<br>4, 13<br>4, 13<br>4, 11<br>4, 11	Recorded neurons (FS, GC)	R_{output}<br>Mean +/- SEM<br><br>0.26±0.05 | 0.12±0.02<br>0.34±0.04 | 0.17±0.02<br>0.40±0.04 | 0.18±0.02<br>0.42±0.04 | 0.21±0.02<br>0.45±0.04 | 0.23±0.02	0.0122<br>0.0001<br>0.0007<br>0.0181<br>0.0307	t(15) = -3.19<br>t(15) = -3.44<br>t(15) = -5.10<br>t(13) = -6.48<br>t(13) = -3.75	R_{input} = 0.88<br>0.84<br>0.74<br>0.65<br>0.56

3A4	one-sample T-test on difference between R_{output} of simultaneously recorded GC and FS	3<br>3<br>3<br>3<br>3	Pairs of recorded neurons	Mean +/- SEM<br>-0.4038 ± 0.02<br>-0.3443± 0.03<br>-0.3266± 0.03<br>-0.1683± 0.002<br>-0.2397± 0.007	0.0024<br>0.0079<br>0.0061<br>0.0002<br>0.0008	t(2) = -20.23<br>t(2) = -11.18<br>t(2) = -12.74<br>t(2) = -78.87<br>t(2) = -35.94	R_{input} = 0.88<br>0.84<br>0.74<br>0.65<br>0.56
3B3	ANCOVA	18, 22	recording sets (HMC, GC)	Linear best fit, 95% CI	0.15	F(2,36) = 1.97	
3B3	Unbalanced two-way ANOVA	18, 22	recording sets (HMC, GC)		0.0004<br>0.074<br>0.57	F(2, 34) = 9.76<br>F(1, 34) = 3.39<br>F(2, 34) = 0.58	Input groups<br>Cell types<br>Interaction
3B3	Post-hoc two-sample T-test with Bonferroni correction for 3 comparison groups	10, 7<br>8, 5<br>4, 6	Recorded neurons (HMC, GC)		1<br>0.05<br>0.21	t(15) = -0.24<br>t(11) = -2.81<br>t(8) = -2.09	R_{input} = 0.76<br>0.26<br>0.11

4D	ANCOVA	15, 22	recording sets (CA3, GC)	Linear best fit, 95% CI	0.0083	F(2,33) = 5.49	
4D	Unbalanced two-way ANOVA	15, 22	recording sets (CA3, GC)		<0.0001<br>0.0036<br>0.24	F(1, 33) = 118.21<br>F(1, 33) = 9.82<br>F(1, 33) = 1.45	Input groups<br>Cell types<br>Interaction
4D	Post-hoc two-sample T-test with Bonferroni correction for 2 comparison groups	6, 11<br>9, 11	Recorded neurons (CA3, GC)		0.032<br>0.1	t(15) = 2.14<br>t(18) = 2.65	R_{input} = 0.76<br>0.11
5C	ANCOVA	200, 610	Recordings for all pairs of input spiketrains (FS, GC)	Linear best fit	<0.0001<br><0.0001<br><0.0001<br><0.0001	F(2,806)=364.8<br>F(2,806)=59.32<br>F(2,806)=66.36<br>F(2,806) = 56.70	Timescale =<br>10ms<br>50ms<br>100ms<br>250ms

5D	ANCOVA	180,<br>220	Recordings for all pairs of input spiketrains (HMC, GC)	Linear best fit	<0.0001<br><0.0001<br><0.0001<br><0.0001	F(2,396)=15.10<br>F(2,396)=21.34<br>F(2,396)=19.33<br>F(2,396) = 24.30	Timescale =<br>10ms<br>50ms<br>100ms<br>250ms
5E	ANCOVA	150,<br>220	Recordings for all pairs of input spiketrains (CA3+gzn, GC+gzn)	Linear best fit	<0.0001<br><0.0001<br><0.0001<br><0.0001	F(2,366)=33.47<br>F(2,366)=17.52<br>F(2,366)=34.20<br>F(2,366) = 108.9	Timescale =<br>10ms<br>50ms<br>100ms<br>250ms
6B	One-sample T-test	8<br>8<br>13<br>13<br>10<br>13<br>11<br>11<br>8<br>4<br>3	recorded neurons	parabolic best fit	< 0.0001<br>0.0001<br>< 0.0001<br>< 0.0001<br>< 0.0001<br>< 0.0001<br>< 0.0001<br>< 0.0001<br>0.0001<br>0.0042<br>0.1125	t(7) = 11.23<br>t(7) = 7.43<br>t(12) = 12.63<br>t(12) = 16.615<br>t(9) = 8.57<br>t(12) = 17.59<br>t(10) = 13.65<br>t(10) = 17.72<br>t(7) = 8.15<br>t(3) = 7.91<br>t(2) = 2.72	Rinput =<br>1<br>0.95<br>0.88<br>0.84<br>0.76<br>0.74<br>0.65<br>0.56<br>0.47<br>0.26<br>0.11
6C left	ANCOVA	102,<br>102	Recordings	mean +/- SEM	0.33	F(2,200) = 1.09	

6C right	one-sample T-test	8<br>8<br>13<br>13<br>10<br>13<br>11<br>11<br>8<br>4<br>3	recording sets	mean +/- SEM	0.63<br>0.09<br>0.03<br>0.06<br>0.96<br>0.06<br>0.73<br>0.46<br>0.77<br>0.85<br>0.60	t(7) = -0.51<br>t(7) = -1.99<br>t(12) = -2.47<br>t(12) = -2.06<br>t(9) = -0.05<br>t(12) = -2.07<br>t(10) = 0.36<br>t(10) = -0.77<br>t(7) = -0.30<br>t(3) = -0.21<br>t(2) = -0.6	Rinput =<br>1<br>0.95<br>0.88<br>0.84<br>0.76<br>0.74<br>0.65<br>0.56<br>0.47<br>0.26<br>0.11
7E bottom	Monte-Carlo exact test (based on proportion of data points above 0)	800<br>800<br>1300<br>1300<br>1000<br>1300<br>1100<br>1100<br>800<br>400<br>300	GC - Shuffle recording sets		0<br>0<br>0<br>0<br>0.055<br>0<br>0.0009<br>0.0845<br>0.0088<br>0.26<br>0.5867		Rinput =<br>1<br>0.95<br>0.88<br>0.84<br>0.76<br>0.74<br>0.65<br>0.56<br>0.47<br>0.26<br>0.11

7E bottom	Monte-Carlo exact test (based on proportion of means above 0)	100<br>100<br>100<br>100<br>100<br>100<br>100<br>100<br>100<br>100<br>100	GC means - Shuffle means	Mean +/- 95% CI	0<br>0<br>0<br>0<br>0<br>0<br>0<br>0<br>0<br>0.01<br>0.85		Rinput =<br>1<br>0.95<br>0.88<br>0.84<br>0.76<br>0.74<br>0.65<br>0.56<br>0.47<br>0.26<br>0.11
8A	Linear regression F-test	102+20+18+15+22 = 177	Recording sets (GC,FS,HMC,CA3+gzn,GC+gzn)	R^2 = 79.4%	< 0.0001	F(1, 175) = 673.1	
8D	One-sample T-test	8<br>8<br>13<br>13<br>10<br>13<br>11<br>11<br>8<br>4<br>3	Recording sets	PSTH Rout means +/- SEM	0.0013<br>0.0125<br>< 0.0001<br>< 0.0001<br>0.0006<br>< 0.0001<br>< 0.0001<br>< 0.0001<br>0.0002<br>0.0107<br>0.0905	t(7) = 5.17<br>t(7) = 3.335<br>t(12)= 6.85<br>t(12) = 7.65<br>t(9) = 5.12<br>t(12) = 9.05<br>t(10) = 8.08<br>t(10) = 8.32<br>t(7) = 7.05<br>t(3) = 5.69<br>t(2) = 3.095	Rinput =<br>1<br>0.95<br>0.88<br>0.84<br>0.76<br>0.74<br>0.65<br>0.56<br>0.47<br>0.26<br>0.11

S3A	Linear regression F-test	102	recording sets	R^2:<br>20%<br>31%<br>41%	<0.0001<br><0.0001<br><0.0001	F(1,100) =<br>24.5<br>45.1<br>69.8	Delay<br>Jitter<br>SP
S3B	Linear regression F-test	102	recording sets	R^2:<br>5%<br>20%<br>61%	0.02<br><0.0001<br>< 0.0001	F(1,100) =<br>5.8<br>25.6<br>160.0	Delay<br>Jitter<br>SP
S3C	Linear regression F-test	20	recording sets	R^2:<br>11%<br>18%<br>88%	0.15<br>0.06<br><0.0001	F(1,18) =<br>2.2<br>3.9<br>129.8	Delay<br>Jitter<br>SP
S3D	Linear regression F-test	177	recording sets	R^2:<br>35%<br>23%<br>57%	<0.0001<br><0.0001<br><0.0001	F(1,175) =<br>74.3<br>41.0<br>183.8	Delay<br>Jitter<br>SP
S4 top	Wilcoxon rank sum test	20, 61	Recording sets (FS, GC)	Mean +/- SEM	< 0.0001<br><0.0001<br>0.0004	Z = 6.1716<br>Z= 5.6787<br>Z = -3.5212	Delay<br>Jitter<br>SP
S4 bottom	Wilcoxon rank sum test	18,22	Recording sets (HMC, GC)	Mean +/- SEM	0.5773<br>0.0267<br>0.0795	Z = 0.5573<br>Z= -2.2157<br>Z = -1.7535	Delay<br>Jitter<br>SP
S5A	ANCOVA	110, 102	recording sets (Simulation , GC)	Linear best fit	< 0.0001	F(2,208) = 92.7	
S5B	Two-sample T-test corrected for samples	110, 102	recording sets (Simulation , GC)		< 0.0001	T(104) = 15.18	

	with unequal variances						
S6(FS)	Two sample T-test	20, 61	recording sets (FS, GC)	Mean +/- SEM	< 0.0001	T(79) = -8.81	
S6 (HMC)	Two sample T-test	18, 22	recording sets (HMC, GC)	Mean +/- SEM	0.0963	T(38) = -1.70	
S6 (CA3)	Two sample T-test	15, 22	recording sets (FS, GC)	Mean +/- SEM	0.0052	T(35) = 2.98	
S7A (FS)	Wilcoxon rank sum test	20, 61	recording sets (FS, GC)	Mean +/- SEM	< 0.0001	Z = -4.2550	
S7C (FS)	One-sided Wilcoxon rank sum test	20, 61	recording sets (FS, GC)	Mean +/- SEM	< 0.0001	Z = -4.7815	
S7A (HMC)	Wilcoxon rank sum test	18, 22	recording sets (HMC, GC)	Mean +/- SEM	0.0005	Z = -3.4663	
S7C (HMC)	One-sided Wilcoxon rank sum test	18, 22	recording sets (HMC, GC)	Mean +/- SEM	< 0.0001	Z = -4.9732	
S7A (CA3)	Wilcoxon rank sum test	15, 22	recording sets (HMC, GC)	Mean +/- SEM	0.18	Z = 1.3456	

S7C (CA3)	One-sided Wilcoxon rank sum test	15, 22	recording sets (HMC, GC)	Mean +/- SEM	0.74	Z = 0.6499	
S8D	ANCOVA	20, 61	Recording sets (nbFS, GC)	Linear best fit	<0.0001	F(2,77)=30.1	
S8D	Unbalanced two-way ANOVA	20, 61	recording sets (nbFS, GC)		0.002<br><0.0001<br>0.84	F(4, 71) = 4.7<br>F(1, 71) = 55.0<br>F(4, 71) = 0.35	Input groups<br>Cell types<br>Interaction
S8F	ANCOVA	180, 220	Recordings for all pairs of input spiketrains (nbHMC, GC)	Linear best fit	0.0001<br><0.0001<br><0.0001<br><0.0001	F(2,396)=9.4<br>F(2,396)=18.2<br>F(2,396)=9.7<br>F(2,396) = 21.8	Timescale =<br>10ms<br>50ms<br>100ms<br>250ms
